# Recent Advances of L-ornithine Biosynthesis in Metabolically Engineered *Corynebacterium glutamicum*

**DOI:** 10.3389/fbioe.2019.00440

**Published:** 2020-01-09

**Authors:** Xiao-Yu Wu, Xiao-Yan Guo, Bin Zhang, Yan Jiang, Bang-Ce Ye

**Affiliations:** ^1^Jiangxi Engineering Laboratory for the Development and Utilization of Agricultural Microbial Resources, College of Bioscience and Engineering, Jiangxi Agricultural University, Nanchang, China; ^2^Laboratory of Biosystems and Microanalysis, State Key Laboratory of Bioreactor Engineering, East China University of Science and Technology, Shanghai, China

**Keywords:** L-ornithine, *Corynebacterium glutamicum*, metabolic engineering, genetic engineering, fermentation

## Abstract

L-ornithine, a valuable non-protein amino acid, has a wide range of applications in the pharmaceutical and food industries. Currently, microbial fermentation is a promising, sustainable, and environment-friendly method to produce L-ornithine. However, the industrial production capacity of L-ornithine by microbial fermentation is low and rarely meets the market demands. Various strategies have been employed to improve the L-ornithine production titers in the model strain, *Corynebacterium glutamicum*, which serves as a major indicator for improving the cost-effectiveness of L-ornithine production by microbial fermentation. This review focuses on the development of high L-ornithine-producing strains by metabolic engineering and reviews the recent advances in breeding strategies, such as reducing by-product formation, improving the supplementation of precursor glutamate, releasing negative regulation and negative feedback inhibition, increasing the supply of intracellular cofactors, modulating the central metabolic pathway, enhancing the transport system, and adaptive evolution for improving L-ornithine production.

## Introduction

The amino acid market is worth several billion dollars. Hence, the production of amino acids has been an active field of biotechnology research in recent years (Lee and Wendisch, 2017; Li et al., 2017; D'Este et al., 2018). Currently, most amino acids are produced by microbial fermentation, a technique adopted in Japan several decades ago. Microbial fermentation is an eco-friendly technology, which can address the growing concerns about environmental issues and can be used to establish a sustainable economy independent of fossil fuels (Becker et al., 2018; Kogure and Inui, 2018). L-ornithine, a non-protein amino acid, is widely used to improve human health as it is reported to have beneficial effects on the liver and the heart (Acharya et al., 2009; Rathi and Taneja, 2018; Butterworth and McPhail, 2019). L-ornithine is also produced by microbial fermentation using *Escherichia coli, Saccharomyces cerevisiae*, or *Corynebacterium glutamicum* as the microbial cell factory (Mitsuhashi, 2014; Becker and Wittmann, 2015). *E. coli* is a widely used microbe for the development of engineered strains to produce chemicals as it has several advantages, such as rapid propagation, availability of a completely sequenced genome, and ease of genetic manipulation (Sarria et al., 2017; Li et al., 2018). *E. coli* could be engineered for producing L-ornithine by rational modulation of the urea cycle and optimizing the fermentation process (Lee and Cho, 2006). However, *E. coli* synthesizes endotoxin, which is banned in the food and pharmaceutical industries (Okuda et al., 2016). This limits the application of *E. coli* as an ideal host to produce L-ornithine. *S. cerevisiae*, an alternative host, has been identified as a generally recognized as safe (GRAS) strain. *S. cerevisiae* exhibits strong robustness or tolerance to harsh growing conditions and is used for the production of various food and pharmaceutical compounds (Guo et al., 2018; Yu et al., 2018; Luo et al., 2019). Modular metabolic engineering strategies were used to engineer the carbon source transport system, central metabolic pathway, ammonia metabolism, energy supply, and transport of small molecular weight compounds in *S. cerevisiae* to produce L-ornithine (Qin et al., 2015). However, the production titer of L-ornithine was low when *S. cerevisiae* was used as a chassis microorganism and hence, the process could not be scaled up to industrial production. As shown in Table 1, *C. glutamicum* is predominantly used to produce L-ornithine. *C. glutamicum*, a gram positive bacterium, was intensively engineered by mutation breeding to enable utilization of a broad spectrum of carbon sources to produce desired chemical compounds (Jeandet et al., 2018; Becker and Wittmann, 2019; Kim et al., 2019). Recent developments in genetic manipulation tools have enabled further genetic modifications, which can further improve L-ornithine production in *C. glutamicum* by metabolic engineering. In this review, we have summarized the current advances in the metabolic engineering strategies for *C. glutamicum*. Comprehensive information on the modification of *C. glutamicum* metabolic pathways has been provided to improve the production of L-ornithine.

**Table 1:** Characteristic of L-ornithine producing strains developed by metabolic engineered strategies.

**Strains**	**Substrate**	**L-ornithine titer (g/L)**	**yield (g/g substrate)**	**Cultivation**	**Modulations**	**References**
*E. coli*						
SJ7055	Glucose	0.052^*^	ND	Shake flask; batch	Inactivation of *argF, argR, argI, proB*, and *speF*; overexpression of *argA214*	Lee and Cho, 2006
*S. cerevisiae*						
M1dM2qM3e	Glucose	5.1	ND	Bioreactor; fed-batch	Modular pathway rewiring	Qin et al., 2015
*C. glutamicum*						
SJC8514 (pEC-*argCJBD*mut)	Glucose	12.48	ND	Shake flask; batch	Overexpression of NCgl0462 and *argCJBD*mut	Kim D. J. et al., 2015
Cc-QF-4	Glucose	40.4	0.27^*^	Bioreactor; batch	Deletion of *proB* and *argF*; positive mutation E19Y of ArgB; heterologous expression of *argA* and *argE*	Shu et al., 2018
SO26	Glucose	43.6	0.34	Bioreactor; fed-batch	Deletion of *argF, NCgl1221, argR, putP, mscCG2*, and *iolR*; attenuation of *odhA, proB, ncgl2228, pta, cat*, and *pgi*; overexpression of *lysE, gdh, cg3035, pfkA, tkt, argCJBD, glt*, and *gdh2*	Zhang B. et al., 2019
SO29	Xylose	18.9	0.4	Shake flask; batch	heterologous expression of *xylAB* operon from *Xanthomonas campestris*	Zhang B. et al., 2019
CO-9	Glucose	6.1	ND	Shake flask; batch	Attenuate expression of *argF* by insertion of a T4 terminator in the upstream region	Zhang et al., 2018c
YW06 (pSY223)	Glucose	51.5	0.24	Bioreactor; fed-batch	Deletion of *argF, argR*, and *proB*; Reinforcement of the PPP pathway flux; The use of a feedback-resistant enzyme	Kim S. Y. et al., 2015
ΔAPE6937R42	Sucrose/molasses	22/27	ND	Shake flask; batch	Heterologous expression of *sacC* from *Mannheimia succiniciproducens*	Zhang et al., 2015
ORN6	Glucose	20.96^*^	0.524	Shake flask; batch	Deletion of *argF, argR*, and *argG*; overexpression of *argB^*M*^*; attenuation of *pgi*.	Jensen et al., 2015
SJC 8260	Glucose	14^*^	ND	Shake flask; batch	Deletion of *argF, argR*, and *proB*; Blocking gluconate biosynthesis	Hwang and Cho, 2014
ORN1(pVWEx1-*glpFKD*^Eco^)	Glycerol	2.24^*^	0.11	Shake flask; batch	Heterologous expression of *glpF, glpK*, and *glpD* from *E. coli*	Meiswinkel et al., 2013b
ΔAPE6937R42	Glucose	24.1	0.298	Bioreactor; batch	Deletion of *argF, argR*, and *proB*; Adaptive evolution in presence of L-ornithine	Jiang et al., 2013a
ORN1(pEKEx3-*xylA_*Xc*_*-*xylB_*Cg*_*)	Xylose	2.59	ND	Shake flask; batch	Overexpression of *xylA* from *X. campestris* and endogenous *xylB*	Meiswinkel et al., 2013a
SJC 8399	Glucose	13.16	ND	Shake flask; batch	Inactivation of the gluconate kinase gene (*gntK*)	Hwang and Cho, 2012
ORN1 (pVWEx1-*araBAD*)	Arabinose	11.7	0.32	Shake flask; batch	Overexpression of *araBAD* from *E. coli* MG1655	Schneider et al., 2011
SJ8074(pEK-P*_*trc*_*::1469)	Glucose	0.32	ND	Shake flask; batch	Overexpression of *NCgl1469*	Hwang and Cho, 2010
1006Δ*argR*-*argJ*	Glucose	31.6	0.396	Shake flask; batch	Deletion of *argR*; overexpression of *argJ*;	Hao et al., 2016
SJC8043	Glucose	3.3	ND	Shake flask; batch	Supplement of L-proline	Lee et al., 2010

* *These values were not described in the main text of the original reference and thus estimated from the figure or graph*.

## Main Biosynthesis Pathways Of L-Ornithine

**Figure 1:**
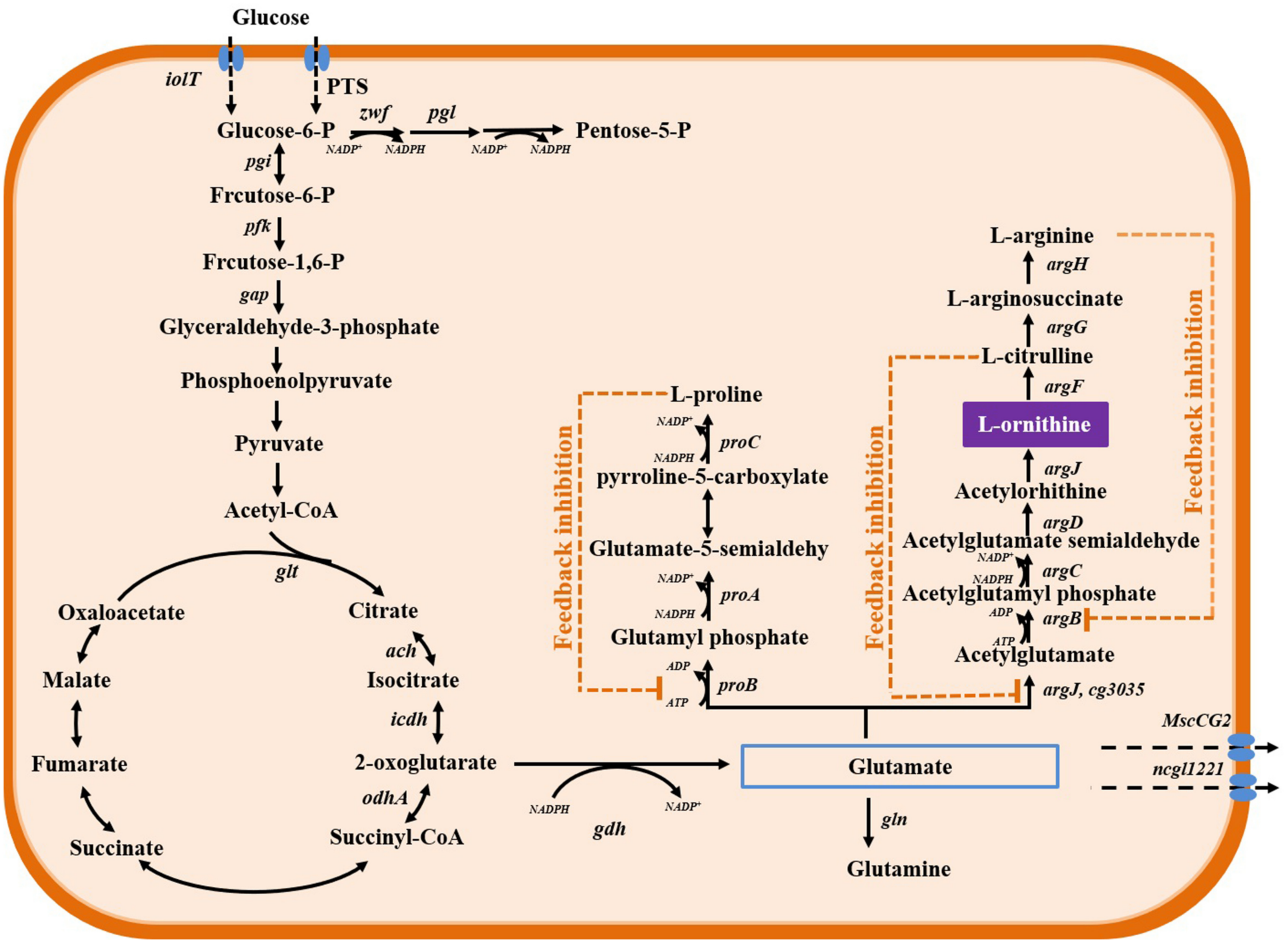
L-ornithine biosynthesis metabolic pathways in *C. glutamicum*. The genes annotation of enzymes as describe in previous study (Zhang B. et al., 2019). The dotted orange line represents feedback inhibition.

**Figure 2:**
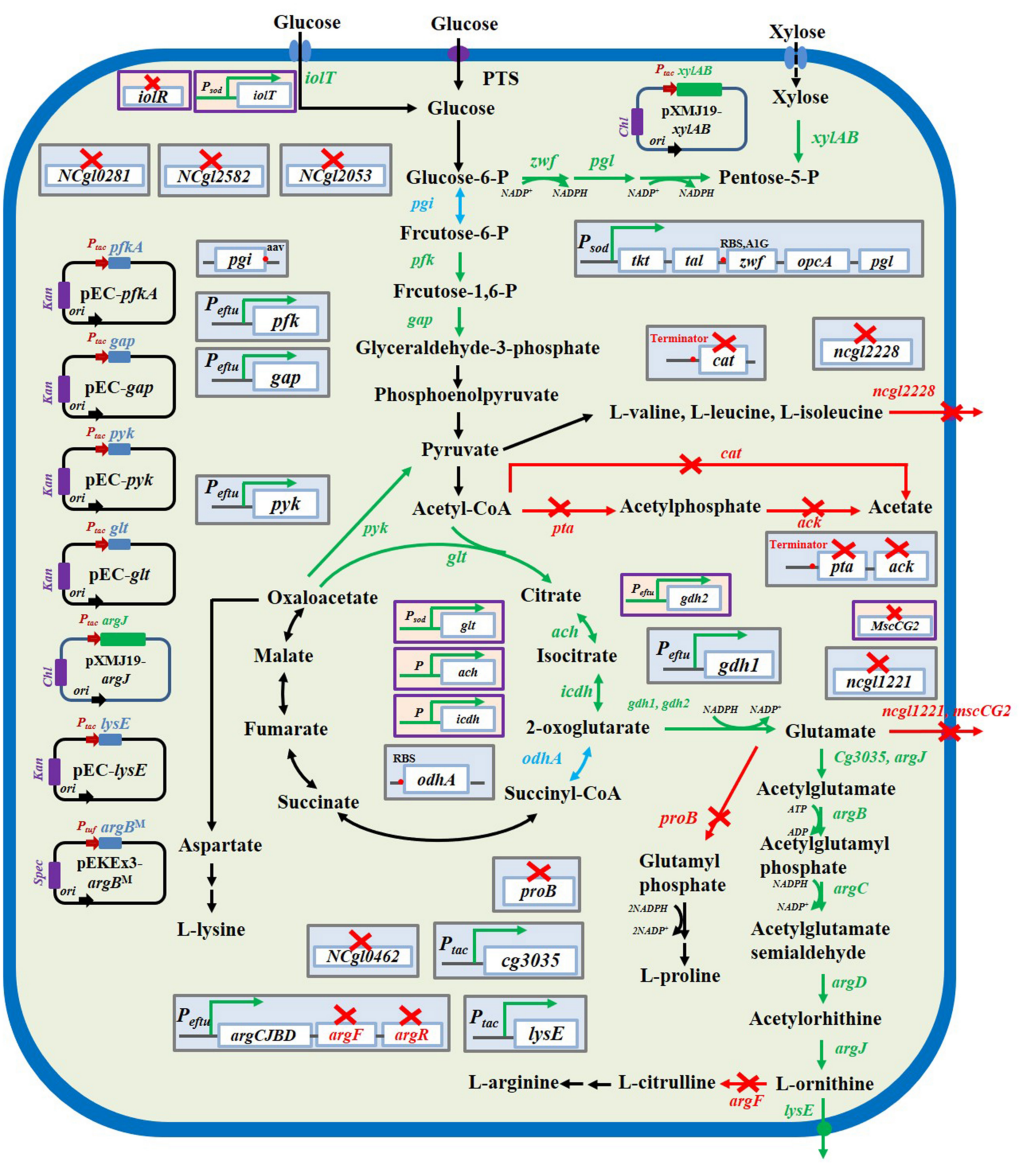
L-ornithine biosynthesis metabolic pathways in *C. glutamicum* and strategies to improve L-ornithine accumulation. The red× represented this pathway were inactivated. The blue font and arrows represented that pathways were attenuated. The green font and arrows indicated that pathways were overexpressed. The genes encoding enzymes involved in catalytically relevant reactions.

In *C. glutamicum*, the biosynthesis of L-ornithine occurs through the urea cycle coupled with the tricarboxylic acid cycle. As described in Figure 1, α-oxoglutarate, an intermediate metabolite in the tricarboxylic acid cycle pathway, can be converted to glutamate by the reversible enzyme, glutamate dehydrogenase (encoded by *gdh*/*NCgl0181*). Further, glutamate is converted to L-ornithine through the enzymatic activities of N-acetylglutamate synthase (encoded by *cg3035*/*NCgl2644*), N-acetylglutamate kinase (encoded by *argB*/*NCgl1342*), N-acetyl-gamma-glutamyl-phosphate reductase (encoded by *argC*/*NCgl1340*), acetylornithine aminotransferase (encoded by *argD*/*NCgl1343*), and ornithine acetyltransferase (encoded by *argJ*/*NCgl1341*). Additionally, L-ornithine can be converted to L-citrulline and L-arginine through the enzymatic activities of ornithine carbamoyltransferase (encoded by *argF*/*NCgl1344*), argininosuccinate synthase (encoded by *argG*/*NCgl1346*), and argininosuccinate lyase (encoded by *argH*/*NCgl1347*). The genes encoding these enzymes are arranged in two operons, *argCJBDFR* and *argGH*. The two operons are repressed by a negative regulatory protein, ArgR/NCgl1345 (Ikeda et al., 2009; Stäbler et al., 2011). The generation of 1 mole of L-ornithine requires 2 moles of NADPH during the conversion of α-oxoglutarate to glutamate and N-acetyl-γ-glutamyl-phosphate to N-acetylglutamate semialdehyde. The enzyme activity of N-acetylglutamate kinase is regulated by L-arginine and L-citrulline via a feedback regulation mechanism. To select and engineer high L-ornithine-producing *C. glutamicum* strains, it is necessary to release the feedback regulation, improve supplementation of cofactor NADPH, and modulate metabolic pathways (Figure 2).

## Blocking The BY-Product Pathways Via Gene Disruption

Under anaerobic conditions, *C. glutamicum* strains are reported to exhibit enhanced glutamate production and decreased L-ornithine production (Gourdon and Lindley, 1999; Lapujade et al., 1999; Hirasawa and Wachi, 2017; Tuyishime et al., 2018). L-ornithine, an intermediate compound in the L-arginine biosynthetic pathway, was synthesized using glutamate as a precursor, which was tightly regulated by feedback inhibition of the terminal metabolites, such as L-arginine and L-citrulline (Ikeda et al., 2009). The enzymatic activity of N-acetylglutamate kinase, which is involved in the main synthetic pathway of L-ornithine, was inhibited by L-arginine and L-citrulline at a concentration of <1 mM (Xu et al., 2012a,b). Thus, the biosynthetic pathways of L-arginine and L-citrulline not only consume L-ornithine, but also hinder its production. To promote the accumulation of L-ornithine, *argF* (encodes ornithine carbamoyltransferase) was disrupted to block the metabolic pathway that converts L-ornithine to L-citrulline and L-arginine. This markedly inhibited the production of L-citrulline and L-arginine in all the L-ornithine-producing *C. glutamicum* strains. However, the deletion of *argF* also resulted in a major hindrance in cell growth as the cells lacked the ability to synthesize arginine. To address this limitation, L-arginine was added to the fermentation medium to meet the demand for cell growth. However, the supplementation of L-arginine increases the cost and the operational complexity. Therefore, it is necessary to adopt appropriate strategies to attenuate the expression of *argF*. Metabolic engineering strategies, such as changing the ribosome binding site (Xiao et al., 2019), replacing the promoter (Xu et al., 2019), changing the translation initiation codon (Otten et al., 2015), and transcription interference by clustered regular interspaced short palindromic repeats (CRISPR) (Cleto et al., 2016; Yoon and Woo, 2018; Park et al., 2019) were successfully applied to optimize the flux of the metabolic pathway. The expression of *argF* was attenuated by changing the ribosome binding site and the translation initiation codon. It was difficult to attenuate the catabolism of L-ornithine owing to the enhanced transcription level of the *argCJBDF* operon. Additionally, the expression of *argF* was attenuated by introducing a strong terminator in the upstream region of *argF*. The L-ornithine titer in the engineered *C. glutamicum* was 6.1 g/L, which was 11-fold higher than that in the original strain and 42.8% higher than that in the mutant strain with *argF* deletion (Zhang et al., 2018c). It must be noted that insertion of a strong terminator in the upstream region of *argF* can not only attenuate the expression of *argF*, but also stimulate the expression of *argCJBD* gene clusters, which are required for the synthesis of ornithine. Therefore, attenuation of *argF* expression is a promising strategy for constructing an efficient L-ornithine-producing *C. glutamicum*.

Glutamate is also utilized to form L-proline via three steps of enzymatic reactions (Lee et al., 2010). The biosynthesis of a compete metabolite of L-proline not only consumes carbon sources, but also the NADPH cofactor, which limits L-ornithine production (Wendisch et al., 2016). In various studies, the *proB*/*NCgl2274* (encodes γ-glutamyl kinase) gene has been deleted to construct high L-ornithine-producing strains by blocking the L-proline biosynthetic pathway. The engineered L-ornithine-producing strains that exhibit enhanced L-ornithine production include YW06 (pSY223) (Kim S. Y. et al., 2015) and Cc-QF-1 (Shu et al., 2018). However, the deletion of *argF* in *C. glutamicum* and inactivation of *proB* negatively affected cell growth due to L-proline deficiency. Thus, proline must be added to the culture medium to maintain normal cell growth. However, the addition of proline to the culture medium increases the operational cost and complexity. To address these limitations, recent studies have attenuated the expression of *proB* by inserting a terminator, which improved L-ornithine production without disturbing the cell growth (Zhang et al., 2018a). As suggested in the modulation of *argF*, attenuating the expression of *proB* by inserting a terminator is an efficient strategy for constructing a L-ornithine-producing *C. glutamicum* strain. In addition to the biosynthetic pathway of proline, the biosynthetic pathways of other amino acids, such as valine, isoleucine, and leucine also consume carbon sources, which limits L-ornithine production. Therefore, a recent study reported that deleting the *ncgl2228* gene (encodes a putative branched amino acid transporter) in the engineered *C. glutamicum* orn8 strain decreased the yield of L-leucine (from 1.90 to 0.13 g/L), L-valine (from 2.16 to 0.22 g/L), and L-isoleucine (from 1.60 to 0.04 g/L) and increased the yield of L-ornithine (from 19 to 22 g/L) (Zhang et al., 2018a). Since the accumulation of by-products in the fermentation prevents the utilization of carbon source and cofactors for producing valuable compounds, it has been concluded that the inactivation of a corresponding transporter is an efficient strategy to reduce accumulation of by-products and increase production of the desired compound.

## Removal Of Feedback Repression And Feedback Inhibition

In the *C. glutamicum* genome, the genes encoding the enzymes involved in the biosynthesis of L-ornithine are present in the *argCJBD* operon, which is repressed by the negative regulatory protein, ArgR. The expression of *argCJBD* operon is repressed at low level, which is an important rate-limiting factor for the biosynthesis of L-ornithine. Hence, the *argR* gene was deleted in all the engineered L-ornithine-producing *C. glutamicum* strains, which provided a powerful strategy for improving the L-ornithine production titer. Inactivation of *argR* in the *C. glutamicum* 1006 strain improved the yield of L-ornithine (from 20.5 to 28.3 g/L) and yield from 0.256 to 0.354 g/g glucose (Hao et al., 2016). However, the yield of L-ornithine did not improve further upon overexpression of the *argCJBD* operon by employing a plasmid or by inserting a strong promoter as the ArgR repressor was absent (Zhang B. et al., 2017; Zhang et al., 2018a). This suggested that the production of L-ornithine limited by the low expression of the *argCJBD* operon can be addressed by removing the ArgR repressor, which concurred with the results described in the construction of the L-arginine-producing strain (Chen et al., 2014; Park et al., 2014).

As described above, the deletion of *argR* removed the transcription control on the main biosynthetic pathway of L-ornithine in *C. glutamicum*. However, stimulation of the *argCJBD* operon expression is not enough for releasing the negative control of the L-ornithine biosynthetic pathway. This is because of the presence of dual control systems in *C. glutamicum*: feedback repression and feedback inhibition. For feedback inhibition of L-ornithine, the enzyme activity of ornithine acetyltransferase (encoded by *argJ*) was inhibited by 50% by using 5 mM L-ornithine. The overexpression of *argJ* increased the yield of L-ornithine from 28.3 to 31.6 g/L in the engineered *C. glutamicum* 1006 strain (Hao et al., 2016). However, the mechanism underlying the inhibition of ornithine acetyltransferase by L-ornithine has not been elucidated. This mechanism can be an effective target for improving L-ornithine production. The gene encoding the ornithine acetyltransferase enzyme was subjected to site-directed mutagenesis, which did not result in the generation of valuable mutants (Shu et al., 2018). Additionally, the fermentation medium for producing L-ornithine was manually supplemented with a certain amount of arginine that resulted in the feedback inhibition of the rate-limiting enzyme, N-acetylglutamate kinase (encoded by *argB*), which is unfavorable for the accumulation of L-ornithine. Several studies have extensively evaluated the release of the negative effect mediated by the addition of L-arginine. The heterologous expression of *argCJBD* derived from the L-arginine-producing strain, *C. glutamicum* ATCC 21831, in the engineered *C. glutamicum* YW03 strain resulted in the production of 7.2 g/L L-ornithine, which was 2.6-fold higher than the L-ornithine titer produced upon expression of *argCJBD* operon derived from *C. glutamicum* ATCC 13032 in the *C. glutamicum* YW03 strain (2.0 g/L) (Kim S. Y. et al., 2015). These results suggest that the *argB* sequences are inconsistent between *C. glutamicum* ATCC 21831 and *C. glutamicum* ATCC 13032, which resulted in differential L-ornithine production. Furthermore, several studies have been performed recently to discover arginine-insensitive N-acetylglutamate kinase in the engineered L-arginine-producing strains (Ikeda et al., 2009; Xu et al., 2012a,b; Zhang J. et al., 2017). The production of L-ornithine was markedly improved upon overexpression of mutant *argB*, such as *argB*^E19R^, *argB*^H268N^, *argB*^G287D^, *argB*^A49V, M54V^, *argB*^A49V, M54V, G287D^, *argB*_*E*.*coli*_ in the engineered *C. glutamicum* ORN2B strain. The L-ornithine titers in the *C. glutamicum* ORN2B (pEKEx3-*argB*^E19R^), ORN2B (pEKEx3-*argB*^A49V, M54V^), and ORN2B (pEKEx3*argB*_*E*.*col*_) strains were 0.307, 0.3, and 0.3 g/g glucose, respectively. These titers were higher than those in the *C. glutamicum* ORN2B (pEKEx3-*argB*) strain (0.257 g/g glucose) (Jensen et al., 2015).

## Improving The Supplementation Of Glutamate By Overexpression Of *gdh* And Blocking Glutamate Secretion

In addition to the optimization of L-ornithine biosynthetic pathway by blocking other competitive metabolic pathways and releasing feedback regulation, adequate supply of precursor glutamate was reported to be another key factor for developing high L-ornithine-producing strains. Currently, many powerful strategies, such as overexpression of the endogenous or exogenous glutamate dehydrogenase, inhibition of the glutamate secretion system, and attenuation of α-ketoglutarate dehydrogenase, have been employed to engineer *C. glutamicum* for improving the flux of glutamate supplementation. The plasmid-based overexpression of *gdh* (encodes glutamate dehydrogenase) in the *C. glutamicum* ΔAP strain increased L-ornithine production by 16% (from 8.6 to 10.0 g/L). Simultaneously, individual chromosomal integration of the *rocG/BSU37790* gene (encodes NADH-coupled glutamate dehydrogenase) derived from *Bacillus subtilis* improved L-ornithine concentration from 12.12 ± 0.57 to 14.84 ± 0.57 g/L in the engineered *C. glutamicum* ΔAPER strain (Jiang et al., 2013b). Additionally, glutamate was synthesized from α-ketoglutarate, which is an intermediate metabolite of the tricarboxylic acid cycle. However, the tricarboxylic acid cycle is closely related to cell growth and cannot be blocked. To balance the cell growth and L-ornithine production, the expression of *odhA* (*NCgl1084*), which encodes an enzyme component of the α-ketoglutarate dehydrogenase complex, was attenuated to reduce the metabolic flux of the tricarboxylic acid cycle and provide more glutamate for the biosynthesis of L-ornithine. When the native ribosome binding site (predicted translation initial intensity was 1,613 au) of *odhA* was replaced with a synthetic ribosome binding site with a predicted translation initial intensity of 837 au on the chromosome, the L-ornithine production titer increased from 13.7 to 16 g/L in the engineered *C. glutamicum* Sorn4 strain (Zhang B. et al., 2017). Furthermore, the overexpression of pyruvate carboxylase and deletion of phosphoenolpyruvate carboxykinase also improved glutamate concentration in the fermentation medium, but did not promote the accumulation of L-ornithine (Hwang et al., 2008). The authors claimed that the supply of glutamate is not a rate-limiting step for L-ornithine production. However, the major reason for the unchanged concentration of L-ornithine may be the extracellular secretion of glutamate, which increased the cost of downstream reaction and precursor wastage. The secretion of glutamate can be inhibited by deleting the *NCgl1221* gene (encodes a glutamate transporter). This was first discovered for developing the L-arginine-producing *C. glutamicum* strain. The deletion of *NCgl1221* markedly improved the L-arginine production titer (Park et al., 2014; Chen M. et al., 2015). Inspired by these reports, inactivation of *NCgl1221* was introduced into the *C. glutamicum* Sorn1 strain, which dramatically decreased the secretion of glutamate and improved L-ornithine production by 22.7% (from 7.97 to 9.8 g/L). However, the deletion of *NCgl1221* is not enough to interrupt the glutamate secretion due to the presence of another glutamate transporter MscCG2 (encoded by *mscCG2*) in *C. glutamicum*. Hence, both *NCgl1221* and *mscCG2* genes were deleted in the engineered *C. glutamicum* S9114 strain, which exhibited negligible glutamate production and enhanced L-ornithine accumulation (Zhang B. et al., 2019). Hence, improving the glutamate availability is an efficient strategy for L-ornithine accumulation in *C. glutamicum*.

## Modulating The Central Metabolic Pathway

Upon consumption of glucose for L-ornithine production by *C. glutamicum* strains, the metabolic flux of central metabolic pathways, including the glycolytic pathway and the tricarboxylic acid cycle pathway, may be a limiting factor as these pathways provide the carbon skeleton. Various strategies have been explored for increasing the metabolic flux of central metabolic pathways for producing L-ornithine. Proteomic studies indicated differential proteomes in the parent strain and L-ornithine-producing strain. The effect of individual plasmid-based overexpression of *pgi* (*NCgl0817*), *pfkA* (*NCgl1202*), *gap* (*NCgl0900*), *pyk* (*NCgl2008*), *pyc* (*NCgl0659*), and *glt* (*NCgl0795*) on L-ornithine production was investigated in the *C. glutamicum* ΔAP strain. Modulating the expression of enzymes involved in the glycolytic pathway, such as Pgi, PfkA, GapA, and Pyk, improved L-ornithine production (Jiang et al., 2013b). A common feature in the enhanced expression of these genes is the improved metabolic flux of glycolysis. Additionally, a recent study focused on strengthening the glycolytic pathway by inserting a strong P_*eftu*_ promoter in the upstream region of *pfkA* in the engineered *C. glutamicum* SO1 strain, which improved the yield of L-ornithine from 23.8 to 26.5 g/L (Zhang et al., 2018b). These results suggested the importance of the glycolytic pathway in L-ornithine production.

The tricarboxylic acid cycle is a source of the carbon skeleton for the biosynthesis of amino acids, such as L-lysine, L-isoline, L-threonine, L-glutamate, L-arginine, L-proline, L-ornithine, and L-citrulline. A recent study reported that rationally engineering the tricarboxylic acid cycle by overexpressing the *pyc, ppc* (*NCgl1523*), and *gdh* genes (encoding pyruvate carboxylase, phosphoenolpyruvate carboxylase, and glutamate dehydrogenase, respectively) and deleting the P1 promoter of *glt* (encodes citrate synthase) improved the lysine yield (from 14.47 ± 0.41 to 23.86 ± 2.16 g/L) and carbon yield (from 36.18 to 59.65%) in the *C. glutamicum* JL68 strain (Xu et al., 2018a). Hence, the tricarboxylic acid cycle pathway was also modulated by overexpressing the *glt, icdh* (*NCgl0634*), and *ach* (*NCgl1482*) genes in the L-ornithine-producing *C. glutamicum* SO16 strain. The insertion of a strong P_*sod*_ promoter in the upstream region of *glt* improved the yield of L-ornithine from 30.8 to 34.1 g/L. Simultaneously, rational modulation of the tricarboxylic acid cycle pathway by overexpressing the *glt* gene improved the glucose consumption in the *C. glutamicum* SO16 strain (Zhang B. et al., 2019).

## Enhanced Supply Of Intracellular Cofactor NADPH And ACETYL-CoA

Efficient supply of the cofactor is important in preventing obstruction of the metabolic pathways to enable production of valuable chemicals using the microbial cell factory (Xu et al., 2018b). NADPH, an important cofactor, is widely used in the biosynthesis of various amino acids, such as lysine (Wu et al., 2019), valine (Zhang H. et al., 2018), methionine (Li et al., 2016), ornithine (Hwang and Cho, 2012), and arginine (Chen M. et al., 2015). Intracellular supplementation of NADPH can be increased by various strategies, such as improving the metabolic flux of the pentose phosphate pathway (Siedler et al., 2013), overexpression of NAD kinase (Lindner et al., 2010; Xu et al., 2016), and developing a NADP-dependent glyceraldehyde 3-phosphate dehydrogenase (Takeno et al., 2010, 2016; Hoffmann et al., 2018). NADPH is a rate-limiting factor in the biosynthesis of metabolites in *C. glutamicum*. In the metabolic pathway of L-ornithine, 2 moles of NADPH are consumed in two steps of enzymatic reactions: conversion from α-oxoglutarate to glutamate catalyzed by glutamate dehydrogenase and from N-acetyl-glutamyl-phosphate to N-acetyl-glutamyl-semialdehyde catalyzed by N-acetyl-γ-glutamyl-phosphate reductase. Ornithine production is frequently limited by the supply of NADPH. Hence, the metabolic flux was redirected toward the pentose phosphate pathway by attenuating *pgi*, replacing the native promoter of *tkt* operon with a strong P_*sod*_ promoter, and changing the translation initiation codon (G1A) of *zwf* (*NCgl1514*) in the YW03 strain, which improved the yield of L-ornithine from 261.3 to 417.2 mg/L (Kim S. Y. et al., 2015). Additionally, the carbon flux of the pentose phosphate pathway was enhanced by deleting the gluconate kinase gene in the *C. glutamicum* SJC8039 strain, which increased the intracellular NADPH concentration by 51.8%. L-Ornithine production yield in the engineered *C. glutamicum* SJC8399 strain with double deletion of *NCgl2399* and *NCgl2905* was 13.16 g/L, which is higher than that (8.78 g/L) in the parent *C. glutamicum* SJC8039 strain (Hwang and Cho, 2012). Furthermore, inactivation of *NCgl0281, NCgl2582*, and *NCgl2053* genes, which encode putative NADP^+^-dependent oxidoreductases, enhanced the intracellular NADPH content by 72.4%. Inactivation of these genes inhibited glucose dehydrogenase activity and improved L-ornithine yield by 66.3% in the engineered SJC8039 strain (Hwang and Cho, 2014). Moreover, NADPH availability for L-ornithine production in the *C. glutamicum* ΔAPER strain was improved by heterologous expression of NADP-dependent glyceraldehyde-3-phosphate dehydrogenase derived from *Clostridium acetobutylicum*, which improved L-ornithine production (Jiang et al., 2013b). Therefore, increased intracellular NADPH supply is a major limiting factor for developing high L-ornithine-producing *C. glutamicum* strains.

In addition to NADPH, the supply of acetyl-CoA cofactor is required to produce L-ornithine. The acetyl-CoA co-factor is consumed during the conversion of oxaloacetate to citrate and glutamate to N-acetylglutamate. Recently, overexpression of *cg3035* (encodes an N-acetylglutamate synthetase) was reported to be an efficient strategy for improving L-ornithine accumulation. This was the first study to report the role of acetyl-CoA in L-ornithine production. The intracellular concentration of acetyl-CoA was improved by attenuating the acetate biosynthesis pathway through insertion of a terminator in the upstream region of *pta*-*ack* (*NCgl2657*-*NCgl2656*) operon or *cat* (*NCgl2480*) in the *C. glutamicum* SO1 strain. Modulation of the acetate biosynthesis pathway improved the yield of L-ornithine from 23.8 to 26 g/L (Zhang et al., 2018b).

## Enhanced Transport System

One of the efficient strategies for developing engineered strains to produce amino acids is overexpression of a specific transport system, such as Ncgl1221 (Nakamura et al., 2007; Nakayama et al., 2012) and MscCG2 (Wang Y. et al., 2018) glutamate transporters, lysine transporter (LysE/NCgl1214) (Vrljic et al., 1996; Kind et al., 2011), phenylalanine transporter (AroP) (Shang et al., 2013), and branched chain amino acid transporters (BrnFE) (Kennerknecht et al., 2002; Chen C. et al., 2015). Currently, there are no reported ornithine-specific transporters. The effect of plasmid-based overexpression of *lysE* (encodes a lysine transporter) on L-ornithine production was investigated in an engineered *C. glutamicum* ATCC 13032 strain containing inactivated *argF*. The intracellular and extracellular concentrations of L-ornithine were measured in short-term fermentation. The analysis indicated that LysE is not an ornithine transporter (Bellmann et al., 2001). However, there was not much progress on the ornithine transport system until the overexpression of *lysE* was studied in the L-ornithine-producing *C. glutamicum* Sorn8 strain. In this strain, the yield of L-ornithine increased by 21.8% when compared to the parent strain after 72 h of fermentation (Zhang B. et al., 2017). Subsequently, the effect of LysE on L-ornithine production was evaluated by deleting the *lysE* gene in the engineered *C. glutamicum* orn1 strain, which reduced the L-ornithine yield by 41.7%. Furthermore, overexpression of LysE by insertion of a strong P_*tac*_ promoter in the upstream region of *lysE* also improved the L-ornithine production titer from 15.3 to 19 g/L. This suggested that LysE promotes L-ornithine production (Zhang et al., 2018a). Although the direct transporter of L-ornithine is not known, the LysE transporter may contribute to developing high L-ornithine-producing strains.

## Adaptive Evolution

The strategies discussed so far for increasing the L-ornithine production titer were based on genetic modifications. However, direct manipulations of one or more genes may negatively affect cell growth or metabolism. Therefore, the combination of irrational metabolic engineering methods, such as adaptive laboratory evolution (Stella et al., 2019) and genetic modification strategies will facilitate the development of high L-ornithine-producing strains. Adaptive laboratory evolution is widely applied for the construction of microbial strains, which not only stimulate latent metabolic pathways but also promote the phenotype and environmental adaptation of engineered strains (Garst et al., 2017; Yu et al., 2018). Compared to rational genetic modifications, adaptive laboratory evolution has provided extensive genetic manipulation targets to improve the strain phenotype. Adaptive laboratory evolution was applied for improving the strain phenotype of *C. glutamicum* ΔAPE. The L-ornithine production titer in the resultant *C. glutamicum* ΔAPE6937 strain was 13.6 ± 0.5 g/L, which was 20% higher than that in the parent strain (11.3 ± 0.3 g/L) (Jiang et al., 2013a). The accelerated cell growth caused by adaptive laboratory evolution was the main factor that contributed to the enhanced L-ornithine production titer in the *C. glutamicum* ΔAPE strain.

## Alternative Substrates

Currently, majority of L-ornithine is produced from glucose, which is also used for human nutrition and animal feed industries. To minimize the competition for glucose between food security and industrial biotechnology, several studies have investigated the production of L-ornithine using non-edible feedstocks, such as xylose and arabinose.

Xylose is the second most abundant sustainable raw material for fermentation. Xylose is obtained from the hydrolysis of lignocellulosic biomasses and is widely used for the production of valuable chemicals, such as succinate (Mao et al., 2018), sarcosine (Mindt et al., 2019), 5-aminovalerate (Jorge et al., 2017b), L-pipecolic acid (Perez-Garcia et al., 2017), 3-hydroxypropionic acid (Chen et al., 2017), and γ-aminobutyric acid (Jorge et al., 2017a) in engineered *C. glutamicum* strains. The utilization of xylose can be improved by heterologous expression of xylose isomerase (Jo et al., 2017), overexpression of myo-inositol/proton symporter IolT1 (Brusseler et al., 2018), and adaptive laboratory evolution (Radek et al., 2017) of *C. glutamicum*. Previously, L-ornithine was produced from xylose by engineering the xylose metabolic pathways in the *C. glutamicum* ORN1 strain. The yield of L-ornithine was further enhanced by overexpressing *xylA* (encodes xylose isomerase) derived from *Xanthomonas campestris* and endogenous *xylB* (encodes xylulokinase) in the *C. glutamicum* ORN1 strain, which improved the L-ornithine yield from 9.4 ± 1.4 to 19.6 ± 1.9 mM (Meiswinkel et al., 2013a). Recently, heterologous expression of *xylAB* operon derived from *X. campestris* in the engineered *C. glutamicum* SO26 strain resulted in the production of 16.32 g/L L-ornithine with a yield of 0.14 g/g xylose. The production titer of L-ornithine was further improved to 18.9 g/L by optimizing the concentration of the inducer, isopropyl β-D-1-thiogalactopyranoside (IPTG) (Zhang B. et al., 2019). Arabinose is another component of lignocellulosic biomass hydrolysate and is widely used for producing valuable compounds (Zahoor et al., 2012; Zhao et al., 2018). Several studies have developed superior microbial cell factories, such as *C. glutamicum* that can efficiently utilize arabinose (Henke et al., 2018; Kawaguchi et al., 2018; Mindt et al., 2019). The heterologous expression of *araBAD* operon derived from *E. coli* enabled the *C. glutamicum* ORN1 strain to utilize arabinose. This engineered strain produced 89 ± 3 mM L-ornithine with a yield of 0.37 M/M arabinose (Schneider et al., 2011).

## Conclusions And Perspectives

Currently, several comprehensive and rational genetic strategies have been employed for engineering *C. glutamicum* strains to produce L-ornithine. There is rapid progress in improving strain performance by combining the optimization of the metabolic pathways in *C. glutamicum*. For example, Kim S. Y. et al. (2015) reported the construction of a recombinant *C. glutamicum* by disrupting *proB, argR*, and *argF*, and overexpressing the operon of *argCJBD* from *C. glutamicum* ATCC 21831. Fed-batch culture of the engineered strain in a 6.6-L fermenter yielded 51.5 g/L of L-ornithine production titer from glucose. In our previous work, a recombinant *C. glutamicum* with modulation in the deletion of *argF, NCgl1221, argR, putP, mscCG2*, and *iolR*, attenuation of *odhA, proB, ncgl2228, pta, cat*, and *pgi*, and overexpression of *lysE, gdh, cg3035, pfkA, tkt, argCJBD, glt*, and *gdh2* produced 43.6 g/L of L-ornithine during a 72 h fed-batch cultivation (Zhang B. et al., 2019). Although the production of L-ornithine by microbial fermentation is a promising and attractive strategy, very few microbes have been used for industrial production due to low yield and low productivity. Metabolic engineering of *C. glutamicum* is an efficient strategy to improve L-ornithine production. The mechanisms underlying the transportation of L-ornithine must be further investigated by new engineering techniques, such as genomics (Teusink and Smid, 2006), transcriptomics (Kim et al., 2017), and proteomics (Shen et al., 2016; Varela et al., 2018) to aid the development of high L-ornithine-producing strains. CRISPR/Cas9, a novel gene-editing technology, is a rapid and efficient method that can be applied for genetic modifications, such as gene deletion, large fragment DNA assembly, or site-directed gene mutation (Cho et al., 2017; Jiang et al., 2017; Wang B. et al., 2018; Zhang J. et al., 2019) in *C. glutamicum*, which can accelerate the pathway engineering in *C. glutamicum* strains. Irrational breeding strategies such as induced mutation breeding, or adaptive evolution have enabled rapid progress in improving the yield of L-ornithine. However, random mutations and labor-intensive screening methods remain as bottleneck issues. Recently, high-throughput screening of mutant strains was developed using a metabolite biosensor, which can measure the metabolite concentrations based on the fluorescence signals or antibiotic selective pressure (Hammer and Avalos, 2016; Lin et al., 2017; Yeom et al., 2018). Due to an improvement in the screening efficiency using a metabolite biosensor, the traditional irrational breeding strategy is considered a promising method for the construction of engineered strains (Zheng et al., 2018). Future studies must focus on developing an efficient intracellular L-ornithine biosensor, which can activate the irrational breeding of high L-ornithine-producing strain.

## Author Contributions

X-YW planned and wrote the first manuscript. X-YG, YJ, and B-CY modified this manuscript. BZ supervised and finalized the manuscript. All authors read and approved the final manuscript.

### Conflict of Interest

The authors declare that the research was conducted in the absence of any commercial or financial relationships that could be construed as a potential conflict of interest. The handling Editor declared a shared affiliation, though no other collaboration, with one of the authors B-CY.
